# Evaluation of free-radical quenching properties of standard *Ayurvedic *formulation *Vayasthapana Rasayana*

**DOI:** 10.1186/1472-6882-11-38

**Published:** 2011-05-12

**Authors:** Sourav Mukherjee, Nayana Pawar, Omkar Kulkarni, Bhagyashri Nagarkar, Shrikant Thopte, Akshay Bhujbal, Pankaj Pawar

**Affiliations:** 1 Modern College for Arts, Science and Commerce, Shivajinagar, Pune - 411 005, Maharashtra, India; 2 Rajiv Gandhi Institute for IT and Biotechnology, Bharati Vidyapeeth University, Pune-Satara Road, Pune - 411 046, Maharashtra, India; 3 Interactive Research School for Health Affairs, Bharati Vidyapeeth University, Pune-Satara Road, Pune - 411 043, Maharashtra, India

## Abstract

**Background:**

Cellular damage induced by free-radicals like Reactive Oxygen and Nitrogen Species (ROS and RNS) has been implicated in several disorders and diseases, including ageing. Hence naturally occurring anti-oxidant rich-herbs play a vital role in combating these conditions. The present study was carried out to investigate the *in vitro *free-radical quenching capacity of a known *Ayurvedic *poly-herbal formulation called *Vayasthapana Rasayana.*

**Methods:**

Methanol extracts of *Vayasthapana Rasayana *formulation (VRF) were studied for *in vitro *total antioxidant activity along with phenolic content and reducing power. *In vitro *assays like DPPH, FRAP, ABTS scavenging to evaluate radical quenching potential were performed.

**Results:**

The formulation has shown 94% at 0.1 mg/ml DPPH free-radical scavenging activity as against 84% at 0.1 mg/ml for standard ascorbic acid (IC_50 _value 5.51 μg/ml for VRF and 39 μg/ml for standard). It has a significant higher ferric reducing potential also (OD 0.87 at 700 nm & 0.21 at 0.1 mg/ml for VRF and standard, respectively). The total phenolic content (gallic acid equivalent) of the VRF is 8.3 mg per g of dry mass. Total antioxidant capacity of the formulation, estimated by FRAP was 1150 ± 5 μM Fe(II)/g dry mass. ABTS radical scavenging activity of VRF was 69.55 ± 0.21% at 100 μg/ml concentration with a IC_50 _value of 69.87 μg/ml as against 9% and 95% by ascorbic acid and Trolox (at 70.452 μg/ml and 0.250 μg/ml concentrations, respectively).

**Conclusion:**

In Indian traditional *Ayurvedic *system, use of VRF is in regular practice for mainly combating age-related disorders and diseases as many of the components of the *Rasayana *are known for their free-radical scavenging activity. This study has validated the potential use of VRF as an anti-oxidant to fight age-related problems.

## Background

In 2006, global aged population (≥ 60 years) numbered 700 million, and it is speculated that by 2050, this number would rise up to 2 billion. Worldwide the population of older individuals is growing at a rate of 2.6% per year, as against the population as a whole (1.1% annually). India stands second in possessing maximum aged population in the world [1]. Besides, deterioration of quality of life due to untimely ageing in the world population needs to be addressed and a suitable solution, either in the form of medication or dietary supplement needs to be explored. At the same time, existing traditional anti-ageing formulations also need to be validated using modern scientific technology and proper explanation.

Reactive Oxygen Species (ROS) and Reactive Nitrogen Species (RNS) including peroxides, super-oxides, hydroxyl radicals and nitrous oxide, generated in the living organisms by cellular metabolism, are known to play a vital role in oxidative cellular damage. Oxidative stress, resulting from these free-radicals plays an important role in manifesting various disorders, including ageing and diseases like cancer, CVD, Parkinson's and in living beings [2,3]. Recent investigations have shown that the antioxidant properties of plants could be correlated with oxidative stress defense, different human diseases and aging process [4]. ROS can easily initiate lipid peroxidation of the membrane lipids, causing damage to the cell membrane composed of phospholipids and lipoproteins by propagating a chain reaction cycle [5]. Thus, antioxidant defense systems have coevolved with aerobic metabolism to counteract oxidative damage from ROS. Also in this regard, total phenol content has received the greatest attention due to the fact that there is a direct relation between total phenol content of the plant with its free-radical scavenging potential [6].

Indian traditional Ayurved system has very specialized therapies for both timely and untimely ageing. According to *Ayurved*, ageing (*Jara*) is as natural as sleep, hunger, or any other instincts. It is described as a natural and inevitable process as well as natural disease [7]. Ageing is characterized by deterioration of natural physical and mental strengths and capabilities. A variety of drugs, selected from plants, minerals and of animal origin, classified under the name *Rasayana*, are in regular use of all Ayurvedic practitioners for *slow down *or *reverting back *timely as well as untimely ageing. These drugs may be having specific actions on respiratory system, cognition etc. or non-specific actions for overall age-related changes.

*Rasayana *therapy is one of the major methods of preservation of health and delaying the process of ageing as described in *Ayurvedic *system of medicine. Good health, according to *Ayurved *as well as modern medicinal systems, means more than being physically healthy. It involves maintenance of subtle functions of the body like cognition, intellect, strength and immunity etc. as observed from the definition of *Rasayana Tantra *(science of rejuvenation and restoration) by Sushuruta (1500 BC) [8]:

Rasayana tantra nama vayasthapanaayurmedhabalakaaram rogaapaharanasamartha cha. Su. Su. 1:7.

This means *Rasayana *has mainly got five different actions; (i) *vayasthapana *delaying the process of ageing, (ii) *aayuskara *i.e. increase in the life span (iii) *balakara *i.e. having anabolic properties to strengthen the body, (iv) *medha balakara *i.e. improvement of cognitive ability, (iv) *roga-apaharana *i.e. gaining immunity and curing from diseases.

The ancient text of *Ayurveda *'*Charaka Samhita' *(1500 BC) explains the component of a standard formulation used for retarding the untimely ageing process, called *Vayasthapana Rasayana *formulation (VRF). This formulation consists of plants, namely *Asparagus racemosus *(*Shatavari*), *Boerhaavia diffusa *(*Punarnava*), *Clitoria ternatea *(*Gokarna*), *Phyllanthus emblica *(*Amla*), *Terminalia chebula *(*Hirda*), *Centella asiatica *(*Mandookparni*) and *Tinospora cordifolia *(*Gulvel*) [8].

In the present study, *in vitro *free-radical scavenging activities of VRF, as a probable basis of combating ageing and age-related disorders, were evaluated.

## Methods

### Chemicals

All chemicals used for assays were of analytical grade. 2,2-diphenyl-1-picrylhydrazyl (DPPH), ABTS (2, 2'-azino-bis (3-ethylbenzthiazoline-6-sulphonic acid), TPTZ (2, 4, 6-tripyridyl-s-triazine), Trolox (6-hydroxy-2,5,7,8-tetramethylchroman-2-carboxylic acid) and gallic acid were procured from Sigma-Aldrich, USA. Potassium persulfate (K_2_S_2_O_8_), sodium nitroprusside (SNP), Ferric chloride (FeCl_3_·6H_2_O), Hydrochloric acid (HCl), potassium hexacyanoferrate (K_2_Fe(CN)_6_), Ferrous sulphate (FeSO_4_) and Tricarboxylic acid were procured from Qualigens Pvt. Ltd, Mumbai, India.

### Plant materials

Fine powders of *Phyllanthus emblica *(*Awla*) and *Boerhaavia diffusa *(*Punarnava*) were procured from Green pharmacy, Pune. Mature stem of *Tinospora cordifolia *(*Gulvel*), unripe fruits of *Terminalia chebula *(*Hirda*), entire plant of *Clitoria ternatea *(*Gokarna*), leaves of *Centella asiatica *(*Mandookparni*) and mature roots of *Asparagus racemossus *(*Shatavari*) were collected from wild sources of Western *Ghats*, dried in shade at 30°C and ground into fine powder.

### Formulation (VRF) preparation

Standard *Vayasthapana Rasayana *formulation (VRF) was prepared by mixing the fine powder of all plants in equal proportion [8].

### Extraction

Methanolic extracts of VRF were prepared by mixing 10% powder in solvent by constant agitation on a shaker (150 rpm, 30°C, 24 h) [9]. Extracts were filtered through Whatman filter paper (No. 1) and the filtrate was centrifuged (10000 rpm, 10°C, 10 min) to obtain a clear supernatant. Its yield was determined and 10 mg/ml stock solution prepared, which was stored in amber coloured bottles at 4°C till further studies.

### Standards

Ascorbic acid was used as a standard for DPPH-free radical scavenging assay and for reducing power assay. Trolox and ascorbic acid served as two standards for ABTS scavenging assay and ferrus sulphate for FRAP assay. For total phenol content calculation, gallic acid was used as the standard.

### *In vitro *anti-oxidant assays

#### Total anti-oxidant capacity (ABTS assay)

The method of Re *et al*. [10] was adopted for the determination of ABTS activity of the formulation (VRF). This assay is based on decolorization that occurs when the radical cation ABTS^.+ ^is reduced to ABTS'(2, 2'-azino-bis (3-ethylbenzthiazoline-6-sulphonic acid). In brief, the radical was generated by reaction of a 7 mM solution of ABTS in water with 2.45 mM potassium persulphate (K_2_O_8_S_2_) (1:1). The mixture was held in darkness at 27°C for 16 h (time needed to obtain stable absorbance at 734 nm). After incubation, the radical solution was further diluted with water (1 ml of ABTS reagent + 27 ml DW) until the initial absorbance value of 0.7 ± 0.005 at 734 nm was reached.

For the assay of test samples 980 μl of ABTS^.+ ^reagent was mixed with 20 μl of the sample or standard. Absorbance was taken after 6 min at 734 nm. ΔO.D. was calculated between initial (0 min.) and 6^th ^min. reading. As a standard, ascorbic acid (8.8 μg/ml to 88.0 μg/ml) and Trolox (0.062 μg/ml to 0.312 μg/ml) were used.

The percentage of scavenging inhibition capacity of ABTS^.+ ^of the extract was calculated using the following equation and compared with ascorbic acid and Trolox.

#### Total antioxidant activity (FRAP assay)

A slightly modified method of Benzie and Strain [11] was adopted for the FRAP assay. The stock solutions included 300 mM acetate buffer (3.1 g CH_3_COONa and 16 ml CH_3_OOH, pH 3.6), 10 mM TPTZ (2, 4, 6-tripyridyl-s-triazine) solution in 40 mM HCl, and 20 mM FeCl_3_·6H_2_O solution. This assay involved (i) preparation of fresh FRAP solution by mixing 25 ml acetate buffer, 2.5 ml TPTZ, and 2.5 ml FeCl_3_·6H_2_O, (ii) raising temperature of the solution to 37°C, (iii) allowing plant extracts (150 μL) allowed to react with 2850 μl of the FRAP solution for 30 min in the dark and (iv) taking readings of the coloured product (ferrous tripyridyl triazine complex) at 593 nm. The standard curve was linear between 200 and 1000 μM FeSO_4_. Results are expressed in μM Fe (II)/g dry mass.

#### DPPH free radical scavenging assay

Complementarities of the antioxidant capacity of the formulation was confirmed by the DPPH scavenging assay according to Brand-Williams *et al*. [12] with slight modification. Different concentrations (0.01 to 0.1 mg/ml) of the extracts and ascorbic acid (standard) were thoroughly mixed with 5 ml of methanolic DPPH solution (33 mg/L) in test-tubes and the resulting solution was kept standing for 10 minutes at 37°C before the optical density (OD) was measured at 517 nm. The measurement was repeated with three sets and an average of the reading was considered. The percentage radical scavenging activity was calculated from the following formula:

Where A_0 _was the absorbance of the control and A_1 _was the absorbance in the presence of the samples.

IC_50 _value was determined from the graph obtained using standard ascorbic acid by using the "y = mx + c" formula from the slope of the graph.

#### Reducing power assay

The Fe^3+^-reducing power of the extract was determined by a method described by Hazra *et al*., [13] with slight modification. The assay involved (i) mixing different concentrations (0.01 to 0.1 mg/ml) of the extracts in phosphate buffer (0.2 M, pH 6.6) with potassium hexacyanoferrate (0.1%), (ii) incubation at 50°C for 20 min, (iii) arresting the reaction by addition of 10% tricarboxylic acid (TCA) and distilled water (2.5 ml), (iv) adding FeCl_3 _solution (0.01%) to the upper portion of the reaction mixture, (v) leaving the reaction mixture for 10 min at room temperature for colour development and (vi) measuring absorbance at 700 nm. All tests were performed in triplicate. Ascorbic acid was used as a positive control. A higher absorbance of the reaction mixture indicated greater reducing power.

### Determination of total phenolic content

The amount of total phenolics present in VRF extract was determined using Folin-Ciocalteu (FC) reagent by Hazra *et al*. [14]. A gallic acid standard curve (R^2 ^= 0.9) was used to measure the phenolic content.

## Results and Discussion

### Total anti-oxidant capacity (ABTS assay) and DPPH free radical scavenging

**Figure 1:**
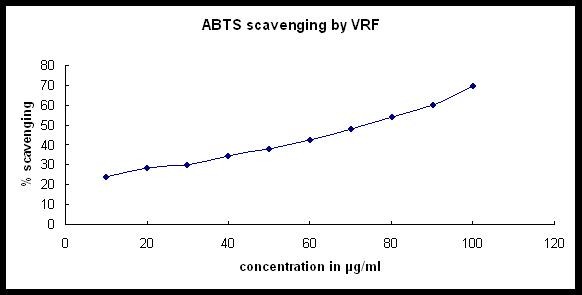
**ABTS free-radical scavenging activity of VRF**.

**Figure 2:**
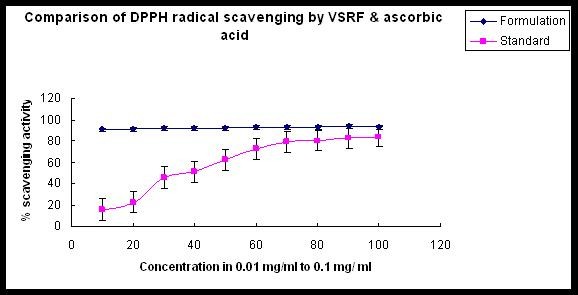
**Comparison of DPPH free-radical scavenging activity of VRF and ascorbic acid**.

The VRF was efficient in scavenging the radical cation ABTS^.+ ^which was reduced to ABTS'. Percentage radical scavenging of the formulation was 69.55 ± 0.21% at a of concentration 100 μg/ml (Figure 1), where that of ascorbic acid and Trolox was 9 ± 0.5% and 95 ± 0.5% at 70.452 μg/ml and 0.250 μg/ml concentration respectively. The IC_50 _value of the formulation was calculated using "y = mx + c" formula, which was 69.87 μg/ml and that for the standard ascorbic acid and Trolox was 0.546 μg/ml and 0.025 μg/ml respectively. VRF was also found to be a potential DPPH-free radical scavenger, since concentration of 0.1 mg/ml, the activity was 94 ± 0.5%. From the graph, the IC_50 _value of the formulation was calculated as 5.51 μg/ml. The percentage scavenging activity of standard (ascorbic acid) was only 84% at 0.1 mg/ml and IC_50 _value was 39 μg/ml (Figure 2).

### Interrelation between DPPH radical and ABTS radical scavenging

Plants with antioxidant activities have been reported to possess free radical scavenging activity [14]. The DPPH scavenging activity in this study indicated that the formulation was potent anti-oxidant. This also suggested that the formulation contained compounds that are capable of donating hydrogen to a free radical in order to remove odd electron, which is responsible for the radical's reactivity [14]. In the present study, standard VRF has shown 93% free radical scavenging activity at a concentration 0.1 mg/ml. It is known that many of these components of *Rasayana *are known as free-radical scavengers [15]. Similarly, *Phyllanthus emblica *and *Terminalia chebula *individually have also shown significantly high DPPH-free-radical scavenging activity (85% and 93%, respectively) at 0.1 mg/ml concentration (data not shown). The free-radical scavenging activity of VRF was more than standard ascorbic acid (84% at 0.1 mg/ml concentration). Also the IC_50 _values of VRF and standard ascorbic acid showed a huge forbidden gap (5.51 μg/ml for VRF and 39 μg/ml for standard), clearly indicating that the formulation is more potent in scavenging free radicals *in vitro*. The ability of this formulation to scavenge DPPH could also reflect its ability to inhibit the formation of ABTS^+^. The scavenging activity of ABTS^+ ^radical by the formulation was found 69.55 ± 0.21% at 100 μg/ml. For DPPH free radical scavenging and ABTS radical scavenging the IC_50 _values were 5.51 μg/ml and 69.87 μg/ml for VRF. This difference in the values may be due to the presence of some potent molecule (s) in the formulation which is more capable of quenching DPPH radical than ABTS radical.

It is known that proton radical scavenging is an important attribute of antioxidants. ABTS, a protonated radical, has characteristic absorbance maxima at 734 nm, which decreases with the scavenging of proton radicals [16,17]. The 2,2'-azinobis-3-ethylbenzothiazoline-6-sulfonic acid (ABTS) activity of the formulation were comparable to standard ascorbic acid and Trolox. This implies that the plant extract may be useful for treating radical-related pathological damage, especially at higher concentration [14]. Since the IC_50 _value of Trolox, which is known to be a potent antioxidant is 0.11 μg/ml, which is significantly low, implies that a very less amount of this antioxidant would give a remarkably high effect in fighting oxidative damage. However, VRF has shown IC_50 _value 69.87 μg/ml, which necessarily indicates that rather a higher dose is required to achieve a desirable effect."

The scavenging of the DPPH radical by the extracts was found to be higher than that of ABTS^+ ^radical. Various factors like (i) stereo-selectivity of the radicals, (ii) solubility of the extract in different testing systems, (iii) polarity of the solvent, (iv) functional groups present in the bioactive compounds, have been reported to affect the capacity of extracts to react and quench different radicals [17]. Our results demonstrated that the formulation under investigation was a potent DPPH-radical and ABTS^+ ^radical scavenger.

### Determination of total phenolic content and FRAP assay

The total phenol content (gallic acid equivalent) of the VRF is 8.3 mg per g of dry mass. It is known that total phenol content is responsible for the free-radical scavenging activities in many plants [6,17]. Total anti-oxidant potential was determined by FRAP and reducing ability of the extracts was 1150 ± 5 μM Fe(II)/g dry mass.

The antioxidant potential of formulations was estimated from their ability to reduce TPRZ-Fe (III) complex to TPTZ-Fe (II). Antioxidant activities are known to increase directly proportional to the poly-phenol content. This activity is believed to be mainly due to their redox properties [17,18], which plays an important role in (a) adsorbing and neutralizing free radicals, (b) quenching singlet and triplet oxygen, and (c) decomposing peroxides [17]. Also according to recent reports, a highly positive relationship between total phenols and antioxidant activity appears to be the trend in many plant species [17]. Our result also shows that the ABTS radical scavenging and DPPH free-radical scavenging activities are significantly high, which may be due to the presence of phenolic compounds in the VRF.

### Reducing power assay

**Figure 3:**
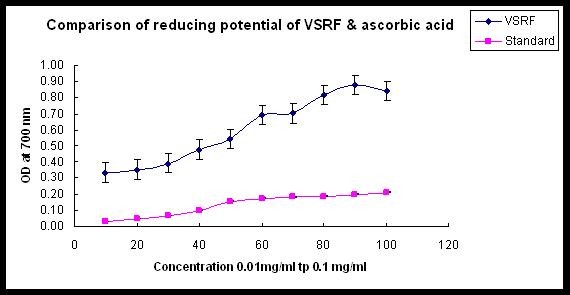
**Comparison of reducing potential of VRF and standard ascorbic acid**.

In the reducing power assay, the presence of antioxidants in the sample would result in the reduction of Fe3+ to Fe2+ by donating an electron. The amount of Fe2+ complex can then be monitored by measuring the formation of Perl's blue at 700 nm. Increasing absorbance indicates an increase in reductive ability [14]. Since reducing power of a compound serves as a significant indicator of its antioxidant activity [19], VRF was assayed for the reducing power activity. It has shown *in vitro *ferric reducing potential. The OD at 700 nm increased in a dose dependent manner from 0.33 at 0.01 mg/ml to 0.87 at 0.1 mg/ml. For this assay also, ascorbic acid was used as a standard. The OD at 700 nm ranged from 0.03 at 0.01 mg/ml and 0.21 at 0.1 mg/ml (Figure 3).

According to Indian Ayurvedic system, ageing may be timely or untimely. Timely ageing, as mentioned by Indian sacred texts, are considered as a natural phenomenon in all living organisms. On the contrary, untimely ageing may be due to faulty food habits, sedentary life-style or stress. Faulty food habits lead to higher degree of accumulation of free radicals in the body. The free-radical theory of ageing emphasizes more on the oxidative stress resulted from cellular metabolism and eventual generation of ROS or RNS [20]. Oxidative stress is also related to apoptosis in unicellular yeasts. It has been demonstrated in yeast, nematodes, flies and primates that mitochondrial ROS production causes cellular damage, resulting into overall decline in cellular functioning, eventually leading to ageing [21].

The ROS and RNS are common free radicals known to damage lipids, proteins, enzymes, and DNA [22,23]. Damages caused by free radicals (hydroxyl radicals, super-oxide anions, hydrogen peroxide, and nitric oxides) lead to cell or tissue injury and a wide range of degenerative diseases, including asthma, ulcer, cancer, Parkinson's [19]. According to free radical theory, as a result of accumulation of oxidatively damaged macromolecules and consequently cells or tissues due to aerobic metabolism to which individuals are continuously exposed, ageing is initiated in human beings [20]. Thus, anti-oxidant defense may be one of the major mechanisms to combat ageing and age-related problems.

## Conclusion

This study affirms the *in vitro *antioxidant potential of crude methanolic extract of the standard *Ayurvedic *formulation, with results comparable or significantly higher to those of the standard compounds such as ascorbic acid and Trolox. Further studies are needed to clarify the *in vivo *potential of the formulation in the management of various age-related human diseases resulting from oxidative stress.

## Competing interests

The authors declare that they have no competing interests.

## Authors' contributions

SM: Prepared the extract, carried out the assays and drafted the manuscript. NMP, BN, ST and AB: Carried out the assays. OPK: Generated the concept and gave Ayurvedic inputs. PKP: coordinated the study. All authors read and approved the final manuscript.

## Pre-publication history

The pre-publication history for this paper can be accessed here:

http://www.biomedcentral.com/1472-6882/11/38/prepub
